# A Signaling Pathway to Mediate the Combined Immunomodulation of Acetylcholine and Enkephalin in Oyster *Crassostrea gigas*

**DOI:** 10.3389/fimmu.2020.00616

**Published:** 2020-04-17

**Authors:** Zhaoqun Liu, Zhi Zhou, Lingling Wang, Yukun Zhang, Yanan Zong, Yan Zheng, Meijia Li, Weilin Wang, Linsheng Song

**Affiliations:** ^1^Liaoning Key Laboratory of Marine Animal Immunology, Dalian Ocean University, Dalian, China; ^2^Functional Laboratory of Marine Fisheries Science and Food Production Processes, Qingdao National Laboratory for Marine Science and Technology, Qingdao, China; ^3^Liaoning Key Laboratory of Marine Animal Immunology and Disease Control, Dalian Ocean University, Dalian, China; ^4^Dalian Key Laboratory of Aquatic Animal Disease Prevention and Control Dalian Ocean University, Dalian, China; ^5^State Key Laboratory of Marine Resource Utilization in South China Sea, Hainan University, Haikou, China

**Keywords:** *Crassostrea gigas*, hemocyte, neurotransmitter, immune regulation, signaling pathway

## Abstract

Molluscs have evolved a primitive but complete neuroendocrine-immune (NEI) system with a vast array of neurotransmitters to conduct both humoral and cellular immunomodulation. Previous studies have illustrated the immune functions of several key neurotransmitters. However, the combined effects of multiple neurotransmitters and the signaling pathway to mediate such immunomodulation have not been well-understood. In the present study, iTRAQ and LC-ESI-MS/MS approaches were employed to investigate the combined immunomodulation functions of two crucial neurotransmitters, acetylcholine (ACh), and [Met^5^]-enkephalin (ENK), in oyster *Crassostrea gigas*. A total number of 5,379 proteins were identified from hemocytes of oysters after the treatments with Ach and ENK separately or simultaneously, and 1,475 of them were found to be significantly up-regulated, while 1,115 of them were significantly down-regulated. The protein expression patterns in the groups treated by ACh and ENK separately were quite similar, which were dramatically different from that in the group treated by ACh+ENK. One hundred seventy-two proteins were found to be differentially expressed in all the three neurotransmitter treatment groups. Functional validation suggested that ACh and ENK possibly modulate the immune response in oyster hemocytes by enhancing pathogen recognition, cell apoptosis, and the enzyme activities of superoxide dismutase (SOD). Moreover, GO enrichment and co-expression network analyses implied that the combined immunomodulation of ACh and ENK might be mediated by p53, EGF-R–ErbB, and Fc gamma R (FcγR) signaling pathways. These results collectively indicated that multiple neurotransmitters executed a combined and ordered immune regulation through common signaling cascades in molluscs, which was under delicate control to maintain the homeostasis.

## Introduction

The hypothesis of neuroendocrine-immune (NEI) regulatory network is proposed on the existence of afferent–efferent pathways between immune and neuroendocrine structures, which refers to a unified feedback network consisting of nervous system, endocrine system, and immune system (1). In vertebrates, NEI network carries a reciprocal regulation role among various systems to maintain the homeostasis with the involvement of signaling molecules, such as neurotransmitters, hormones, and cytokines (1). As the first identified neurotransmitter, acetylcholine (ACh) has been approved to attenuate the synthesis of TNF and IL in macrophages of adult male Lewis rats (2). [Met^5^]-enkephalin is another neurotransmitter of native opioid peptide participating in cell proliferation and immune regulation (3, 4). Unlike vertebrates, molluscs lack adaptive immunity and rely solely on the innate immune system to fight against invading pathogens. Recently, a primitive but complete NEI system has been characterized in molluscs (5–7), which shares structural and molecular similarities with that in vertebrates. As mollusca is the most primitive phyla with a complete NEI system (8), a better understanding of the molecular basis and regulatory pathways of molluscan NEI regulation will undoubtedly contribute to the comparative study of the innate immunity.

It has been demonstrated that neurotransmitters play an important role in the neuroendocrine regulation in molluscs (9, 10). Once the host encounters a threat, such as pathogen invasion or physical stress, the NEI network is activated immediately to synthesize and release neurotransmitters to modulate the immune response (6, 11, 12). Several neurotransmitters had been proved to be critical for the immunomodulation in molluscs, among which ACh and ENK exhibited the most extraordinary functions. It was reported that the ACh in scallop hemolymph could be induced by the treatments with LPS and TNF-α, and the up-regulation of superoxide dismutase (SOD), catalase (CAT), and lysozyme (LYZ) induced by LPS treatment could be significantly inhibited by ACh (13). During the ontogenesis of oyster, the activated enkephalinergic nervous system could regulate the expression of pathogen recognition receptors (PRRs), immune effectors, the anti-bacterial activities, as well as the cytokine concentrations in the larvae of oyster *Crassostrea gigas* (14). The immune regulations of ACh and ENK were also reported to impose combined immunomodulation together in molluscs. After co-treatment of ACh and ENK, the translocation of transcription factor p65 from the cytoplasm into the nuclei was triggered, and the apoptosis index and phagocytosis rate of oyster hemocytes both decreased significantly, suggesting that the activity of ENK to up-regulate the immune response could be overwhelmed by the down-regulation effects of ACh (6). However, the knowledge about the immunomodulation mechanisms of ACh and ENK are only limited to their corresponding receptors on hemocytes, while the exact signaling pathway is still unclear.

In our previous studies, the activation patterns and immune regulation modes of the NEI network in oyster *C. gigas* at mRNA levels were characterized, which provided an entrance to better understand the NEI regulatory network in invertebrates. However, the transcriptional profiling can only contribute partially to the understanding of the neuroendocrine immunomodulation patterns since not all transcripts can be translated and the mRNA abundances do not correspond to the protein level due to pre-, co-, and post-translational modification. In addition, proteins, not mRNA, are the effectors of biological functions (15, 16), and critical regulatory signaling events downstream of transcription will not be detected by transcript analysis (17). In the present study, iTRAQ method combined with LC-ESI-MS/MS was employed to identify the differentially expressed proteins in oyster hemocytes under the stimulation of ACh and ENK with the objectives to (1) identify the molecular components of NEI regulatory network at protein levels in oyster *C. gigas*, (2) investigate the signaling pathways mediating the modulation of ACh and ENK, and (3) evaluate the synergic effects of ACh and ENK on oyster hemocytes.

## Materials and Methods

### Oysters, Treatments, and Sample Collection

Oysters *C. gigas* (averaging 110 mm in shell height) were collected from a local farm in Qingdao, Shandong Province, China, and maintained in the aerated seawater at 20°C for 2 weeks before processing.

Sixty oysters were employed and randomly divided into four groups with 15 individuals in each group. Oysters in all groups received the first injection with 100 μl of lipopolysaccharide (LPS, origin from *Escherichia coli* 0111:B4, Sigma) at a working concentration of 0.5 mg ml^−1^ as described before (18). Six hours post injection, each oyster in the seawater (SW) group received an injection of 100 μl of filtered seawater, while the oysters in the other three experimental groups received injections with 100 μl of ACh (A6625, Sigma Aldrich, 10^−7^ mol L^−1^ in SW, designated as ACh group) (18), [Met^5^]-enkephalin (M6638, Sigma Aldrich, 10^−4^ mol L^−1^ in SW, designated ENK group) (19), and mixture of ACh and ENK (10^−4^ mol L^−1^ ENK and 10^−7^ mol L^−1^ ACh in SW, designated as ACh_ENK group) (6), respectively. After treatment, these oysters were returned to water tanks, and sampled at 6 h post-injection. Three biological replicates were sampled for each group.

Another 60 oysters were employed and randomly divided into four groups with 15 individuals in each group. The oyster in the ENKR^−^ (ENK receptor block) group received the first injection of 100 μl of 7-benzylidenenaltrexone maleate (BNTX, ENKR receptor antagonist, Tocris Bioscience) (19). In the AChR^−^ (acetylcholine receptor block) group, oysters received an injection with 100 μl of a mixture of mecamylamine hydrochloride (non-selective nicotinic AChR antagonist, Tocris Bioscience) and five types of antagonists including pirenzepine (Tocris Bioscience), AFDX 116 (Tocris Bioscience), 4-DAMP (Tocris Bioscience), PD102807 (Tocris Bioscience), and darifenacin (Sigma) specific for the m_1_-m_5_ muscarinic AChRs (mAChRs) (7). In the AChR^−^+ENKR^−^ group, oysters were treated with a mixture of BNTX, mecamylamine hydrochloride, pirenzepine, AFDX 116, 4-DAMP, PD102807, and darifenacin to block both AChRs and ENKRs. The final concentration of each antagonist was 10.0 μmol L^−1^. The oysters in SW group received an injection of seawater. One hour post-injection, the oysters in all groups received an injection with 100 μl of LPS at a working concentration of 0.5 mg ml^−1^, while the oysters in the SW group received another injection of seawater. At 6 h post-injection, each oyster in the SW group received an injection of 100 μl of seawater, while oysters in the other three experimental groups received injections of 100 μl of ACh (10^−7^ mol L^−1^), ENK (10^−4^ mol L^−1^), and a mixture of ACh and ENK (10^−4^ mol L^−1^ ENK and 10^−7^ mol L^−1^ ACh), respectively. After treatment, these oysters were returned to water tanks, and sampled at 6 h post-injection. Three biological replicates were sampled for each group.

Hemolymph from five individuals in each group were pooled together as one parallel and centrifuged at 800 × *g* at 4°C for 10 min to harvest the hemocytes for proteomic analysis. Serum from five oysters were sampled as one duplicate and stored at −80°C for subsequent enzyme activity determination. There were three replicates for each assay. All experiments involving animals reported in this study were approved by the Ethics Committee of the Institute of Oceanology, Chinese Academy of Sciences.

### Protein Preparation

Oyster hemocytes were suspended in the Lysis buffer (7 M urea, 2 M thiourea, 4% CHAPS, 40 mM Tris–HCl, pH 8.5, 1 mM PMSF, and 2 mM EDTA) and sonicated on ice. The proteins were reduced with 10 mM DTT (final concentration) at 56°C for 1 h and then alkylated by 55 mM IAM (final concentration) in the darkroom for 1 h. The reduced and alkylated protein mixtures were precipitated by adding 4 × volume of chilled acetone and kept at −20°C overnight. After centrifugation at 30,000 × *g* at 4°C for 20 min, the pellet was dissolved in 0.5 M TEAB (Applied Biosystems, Milan, Italy) and sonicated on ice. After centrifuging at 30,000 × *g* at 4°C for 10 min, an aliquot of the supernatant was taken for determination of protein concentration. The protein in the supernatant was kept at −80°C for further analysis.

### iTRAQ Labeling and SCX Fractionation

Total protein (100 μg) was extracted from each sample and then digested with Trypsin Gold (Promega, Madison, WI, USA) with the ratio of protein:trypsin = 30:1 at 37°C for 16 h. The obtained peptides were dried by vacuum centrifugation and then reconstituted in 0.5 M TEAB. They were further processed according to the manufacturer's protocol for 8-plex iTRAQ reagent (Applied Biosystems). Briefly, one unit of iTRAQ reagent was thawed and reconstituted in 24 μl of isopropanol. The peptides were labeled with the isobaric tags, incubated at room temperature for 2 h. The labeled peptide mixtures were then pooled and dried by vacuum centrifugation.

SCX chromatography was performed with a LC-20AB HPLC Pump system (Shimadzu, Kyoto, Japan). The iTRAQ-labeled peptide mixtures were reconstituted with 4 ml buffer A (25 mM NaH_2_PO_4_ in 25% ACN, pH 2.7) and loaded onto a 4.6 × 250 mm Ultremex SCX column containing 5-μm particles (Phenomenex). The peptides were eluted at a flow rate of 1 ml min^−1^ with a gradient of buffer A for 10 min, 5–60% buffer B (25 mM NaH_2_PO_4_, 1 M KCl in 25% ACN, pH 2.7) for 27 min, and 60–100% buffer B for 1 min. The system was maintained at 100% buffer B for 1 min and equilibrated with buffer A for 10 min prior to the next injection. Elution was monitored by measuring the absorbance at 214 nm, and the fractions were collected every 1 min. The eluted peptides were pooled into 20 fractions, desalted with a Strata X C18 column (Phenomenex), and vacuum-dried.

### LC-ESI-MS/MS Analysis Based on Exactive

Each fraction was resuspended in buffer A (2% ACN, 0.1% FA) and centrifuged at 20,000 × *g* for 10 min. The final concentration of each peptide was adjusted to about 0.5 μg μl–^1^. An aliquot of 10 μl supernatant was loaded on a LC-20AD nanoHPLC (Shimadzu, Kyoto, Japan) by the autosampler onto a 2-cm C18 trap column. The peptides were eluted onto a 10-cm analytical C18 column (inner diameter, 75 μm) packed in-house. The samples were loaded at 8 μl min^−1^ for 4 min and then the 44-min gradient was run at 300 nl min^−1^ starting from 2 to 35% B (98% ACN, 0.1% FA), followed by 2 min linear gradient to 80%, and maintenance at 80% B for 4 min, and finally returned to 5% for 1 min.

The peptides were subjected to nano-electrospray ionization followed by tandem mass spectrometry (MS/MS) in a Q EXACTIVE (Thermo Fisher Scientific, San Jose, CA) coupled online to the HPLC. Intact peptides were detected in the Orbitrap at a resolution of 70,000. Peptides selected for MS/MS were measured by using high-energy collision dissociation (HCD) operating mode with a normalized collision energy setting of 27.0. The ion fragments were detected in the Orbitrap at a resolution of 17,500. A data-dependent procedure alternating between 1 MS scan followed by 15 MS/MS scans was applied for the 15 most abundant precursor ions above a threshold ion count of 20,000 in the MS survey scan with a following Dynamic Exclusion duration of 15 s. The applied electrospray voltage was 1.6 kV. Automatic gain control (AGC) was used to optimize the spectra generated by the orbitrap. The AGC target for full MS and MS2 was 3e6 and 1e5, respectively. The m/z scan range for MS and MS2 scans ranged from 350 to 2,000 Da and 100 to 1,800 Da, respectively.

### Bioinformatical Analysis

Protein identifications were performed by using Mascot search engine (Matrix Science, London, UK; Version 2.3.02). A mass tolerance was permitted for intact peptide masses, with allowance for one missed cleavage in the trypsin digests. Gln- > pyro-Glu (N-term Q), Oxidation (M), and Deamidated (NQ) were employed as the potential variable modifications, and Carbamidomethyl (C), iTRAQ8plex (N-term), and iTRAQ8plex (K) were employed as fixed modifications. The charge states of peptides were set to +2 and +3. Specifically, an automatic decoy database search was performed in Mascot by choosing the decoy checkbox in which a random sequence of database was generated and tested for raw spectra as well as the real database. To reduce the probability of false peptide identification, only peptides at the 95% confidence interval by a Mascot probability analysis greater than “identity” were counted. For protein quantitation, it was required that a protein contained at least two unique peptides. The database used to identify peptides was Crassostrea_nr (25,994 sequences, http://www.ncbi.nlm.nih.gov/protein?LinkName=genome_protein&from_uid=10758). The quantitative protein ratios were weighted and normalized by the median ratio in Mascot. Only the ratios with *p* < 0.05 were used, and only fold changes of *p* > 1.2 were considered as significant. Functional annotations of the proteins were conducted using Blast2GO program against the non-redundant protein database (NCBI). The KEGG database (http://www.genome.jp/kegg/) and the COG database (http://www.ncbi.nlm.nih.gov/COG/) were used to classify and group these identified proteins.

Co-expression analysis was performed using WGCNA in order to identify the modules of highly correlated proteins (20, 21). CV values were calculated for all proteins, and those with a CV <0.8 across samples were not included in the WGCNA analyses. All FPKM expression values of the proteins were log_2_ transformed, and any log_2_ FPKM values <1 were set to 0. An R version 3.0.1 (http://www.R-project.org/) implementation of the WGCNA package was used to identify gene modules (22) with parameters β and treecut of 20 and 0.9, respectively. All other parameters were set with the default values. Eigenproteins, the average normalized protein expressions for a module, were calculated for each protein co-expression module. The Biological Networks Gene Ontology tool (BiNGO) was employed to assess overrepresentation of Gene Ontology (GO) categories in Biological Networks, and the Benjamini and Hochberg correction was used for FDR correction. Cytoscape v3.0.1 (23) was used to visualize the networks and Photoshop was used to edit the images.

### Determination of Hemocyte Apoptosis and SOD Enzyme Activity

The apoptosis index (AI) of oyster hemocytes was determined according to the manual of Annexin V-FITC Apoptosis Detection Kit (KeyGEN, China). The collected hemocytes (10^6^ in total) were washed twice by sterile seawater, and resuspended in 195 μl of binding buffer. The hemocyte resuspension was incubated with 5 μl of Annexin V-FITC in the dark for 10 min, and resuspended with 190 μl of Binding Buffer after centrifugation at 1,000 × *g* for 5 min. After incubation with 10 μl of propidium iodide at 0°C in the dark for 15 min, the hemocyte resuspension was transferred into a polystyrene round-bottom tube and the AI was determined by flow cytometry (BD FACS Aria II SORP). The average AI of three tubes was calculated as below:

$$\begin{array}{l} AI=\frac{\text{Number of apoptotic cells}}{\text{Number of haemocytes}} \end{array}$$

The activity of immune-related enzyme SOD in oyster serum was measured by the kit (Jiancheng, A001-1, Nanjing) according to the protocols. SOD activity was defined by the hydroxylamine method as the ability of 1 mg protein to cause 50% inhibition in 1 ml of reaction solution. SOD activity was expressed as unit activity per milligram of protein in the sample (U mg prot^−1^, *n* = 3).

### Protein Purification, Polyclonal Antibody Preparation, and Western Blot Assay

Four genes including *Cg*C1qDC-8 (EKC38694.1), *Cg*Galectin-9 (EKC40501.1), extracellular SOD (*Cg*eSOD, EKC39002.1), and programmed cell death protein 4 (*Cg*PCDP4, EKC31180.1), which were differentially expressed in all the three neurotransmitter treatment groups, were selected for the functional validation. Briefly, the PCR products of these genes were gel-purified and cloned into pET-30a expression vector with a His tag. The forward positive clones were screened by PCR and further confirmed by nucleotide sequencing. The valid recombinant plasmid was extracted and transformed into *E. coli* Transetta DE3. Positive transformants were cultured in LB medium at 37°C shaking at 200 rpm. When the culture medium reached OD_600_ of 0.4–0.6, the bacteria were incubated for additional 8 h with the induction of isopropyl β-D-1-thiogalactopyranoside (IPTG) at the final concentration of 1 mM. The recombinant proteins with a six-His (6 × His) tag at the C-terminal was purified by Ni^+^ affinity chromatography and desalted by extensive dialysis.

Six-week-old mice were immunized with the recombinant proteins to acquire polyclonal antibodies. Briefly, 100 μl of recombinant proteins (0.3 mg/ml) was emulsified with 100 μl of complete Freund's adjuvant (Sigma, USA) to immunize each mouse by subcutaneous implantation. The second and third injections were performed on the 14th and 21st day with incomplete Freund's adjuvant (Sigma, USA). The fourth injection was performed with 100 μl of purified protein on the 28th day. The serum containing specific polyclonal antibodies was obtained on the 36th day and stored at −80°C before use. The binding specificity of these polyclonal antibodies was carefully validated.

The protein expression levels of *Cg*C1qDC-8, *Cg*Galectin-9, *Cg*eSOD, and *Cg*PCDP4 in oyster hemocytes under neurotransmitter immunomodulation were verified by Western blot assay. The total protein extract of hemocyte was separated by 12% SDS-PAGE and electrophoretically transferred onto a nitrocellulose membrane. The membrane was washed three times with TBS containing 0.1% Tween 20 (TBST), blocked by 5% skimmed milk (100 ml TBST, 5 g skimmed milk) at 4°C overnight, and then incubated with 1:1,000 diluted polyclonal antibodies at 37°C for 3 h. After three times of washing with TBST, the membrane was incubated with 1:3,000 diluted secondary antibody goat-anti-mouse IgG conjugated with HRP (ABclonal) at 37°C for 2 h. After washing three times with TBST, the protein bands were developed by using Super ECL Detection Reagent (Sigma-Aldrich) for 2 min. Finally, exposure was performed by Amersham Imager 600 system (GE Healthcare, USA).

### Statistical Analysis

Statistical analysis was performed and all data were given as means ± S.D. Statistical significance was determined by two-tailed Student's *t* test, or by one-way analysis of variance (ANOVA) followed by S-N-K *post hoc* test for multiple comparisons. Statistically significant difference was designated at *p* < 0.05, indicated by different letters.

## Results

### The Overall Characterization of the Proteome Data

**Figure 1:**
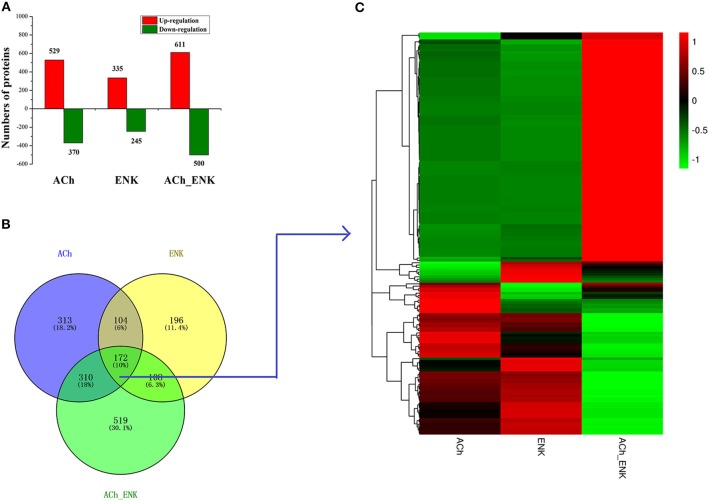
Number of differentially expressed proteins under ACh, ENK, and ACh_ENK treatments. **(A)** A total of 4,956 differentially expressed proteins were identified in all three neurotransmitter treatment groups compared with that in the SW group. **(B)** A Venn diagram showing the common and unique expressed proteins and **(C)** hierarchical clustering of the 172 differentially expressed proteins responsive to all three treatments.

A total of 308,986 spectra were generated from iTRAQ experiments. Based on the Mascot search results, 87,886 spectra matched known spectra, 81,309 spectra matched unique peptides, 26,940 matched peptides, 25,865 matched unique peptides, and 5,379 matched proteins (Table S1). A quantitative strategy by using the mass spectrum data was employed to identify differentially expressed proteins. The differentially expressed proteins were determined by the Mascot software (Matrix Science, London, U.K.; Version 2.3.02) with a screening criterion of 1.2-fold change in abundance (24, 25). A total of 4,956 differentially expressed proteins were identified in all three neurotransmitter treatment groups compared with that in the SW group. Among these differentially expressed proteins, 2,590 proteins were uniquely differentially expressed in ACh, ENK, and ACh_ENK groups, with 1,475 (57.0%) up-regulated and 1,115 (43.0%) down-regulated (Figure 1A). There were 172 proteins differentially expressed in all three experimental groups compared with that in the SW group (Figure 1B and Table S2). The expression patterns of proteins in ACh and ENK groups were quite similar, which was different from that in the ACh_ENK group (Figure 1C).

### GO Term Distributions of the Differentially Expressed Proteins

**Figure 2:**
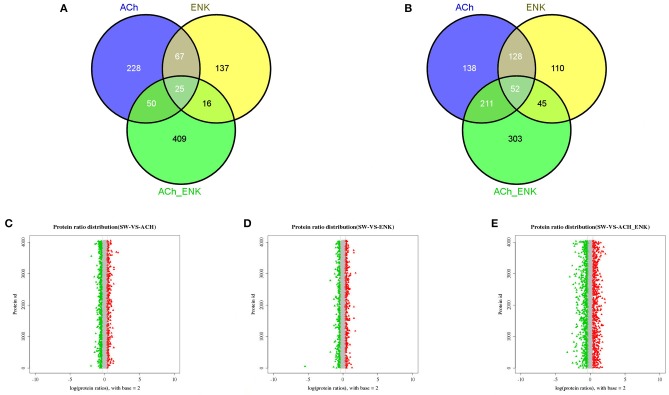
Venn diagrams showing the common and unique up-regulated **(A)** and down-regulated **(B)** expressed proteins, and distribution of differentially expressed protein abundances in ACh **(C)**, ENK **(D)**, and ACh_ENK **(E)** groups. The *x*-axis indicates the fold change (ratios) of proteins based on the logarithm with base 2. The *y*-axis indicates the protein ID. The candidate differentially expressed proteins were indicated in red (up-regulated) or green (down-regulated), with the absolute value of log_2_ (protein fold change) > 1.5.

Compared with that in the SW group, there were 529, 335, and 611 significantly up-regulated proteins, and 370, 245, and 500 significantly down-regulated proteins, identified in ACh, ENK, and ACh_ENK groups, respectively (Figures 2A,B, Table S3). The GO term distribution analysis of those proteins was completed at multiple GO levels (Table S4 and Figures S1–S6). In the lists of the up-regulated proteins between ACh and SW groups, a total of 95 GO terms were characterized (Figure S1), including oxidoreduction coenzyme metabolic process (GO: 0006733), glucose metabolic process (GO: 0006006), and exocytosis (GO: 0006887), etc. Nine GO terms, such as SOD activity (GO: 0004784), oxidoreductase activity, acting on superoxide radicals as acceptor (GO: 0016721), and alpha-amino acid metabolic process (GO: 1901605), were identified in the up-regulated protein lists between ENK and SW groups (Figure S2). While 189 GO terms were identified in the up-regulated protein lists between ACh_ENK and SW groups, including cell motility (GO: 0048870), cellular respiration (GO: 0045333), aerobic respiration (GO: 0009060), etc. (Figure S3).

In the lists of significantly down-regulated protein between ACh and SW groups, a total of 262 GO terms were characterized (Figure S4), including ATP metabolic process (GO: 0046034), unfolded protein binding (GO: 0051082), posttranscriptional regulation of gene expression (GO: 0010608), etc. Meanwhile, 175 GO terms were enriched in the down-regulated protein lists between ENK and SW groups (Figure S5), such as cellular catabolic process (GO: 0044248), phosphotransferase activity, phosphate group as acceptor (GO: 0016776), and cAMP-dependent protein kinase regulator activity (GO: 0008603). There were 262 GO terms identified in the down-regulated protein lists between ACh_ENK and SW groups (Figure S6), including hydrolase activity, acting on acid anhydrides (GO: 0016817), macromolecule catabolic process (GO: 0009057), energy coupled proton transport, down electrochemical gradient (GO: 0015985), etc.

### Functional Annotation and Co-expression of Differentially Expressed Protein

The dynamic range of differentially expressed protein abundances was shown in Figure 2. Potential biomarkers were included in the red and green plots to represent various expression profiles. In the ACh_ENK group receiving a combined regulation of two neurotransmitters, there were more proteins expressed significantly different. This result was consistent with the cluster analysis of the co-differentially expressed proteins (Figure 2E), indicating that the combined treatment of two neurotransmitters could impose much stronger regulation compared with that of single kind of neurotransmitter and even revert the response pattern.

**Figure 3:**
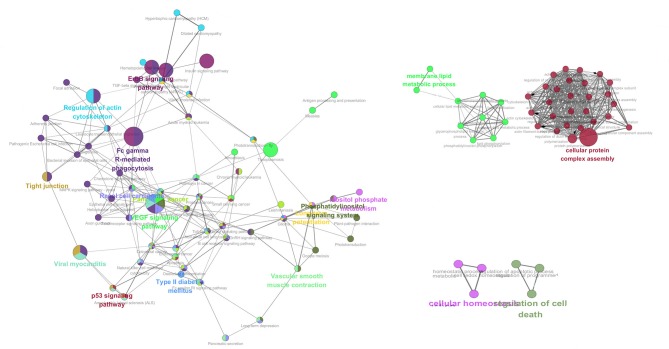
The co-expression network analysis of the enriched GO terms in ACh, ENK, and ACh_ENK treatment groups. The network was constructed with WGCNA package in Rstudio software and illustrated with Cytoscape software.

GO overrepresentation analysis was employed to illustrate the co-expression of significantly expressed proteins after neurotransmitter treatment and the possible neuroendocrine immunomodulation modes of oyster hemocyte at protein levels in response to LPS stimulation. Genes involved in signaling pathways including ErbB signaling pathway, p53 signaling pathway, EGF signaling pathway, Fc gamma R (Fcγ R)-mediated phagocytosis, as well as some key physiological activities such as cellular protein complex assembly, membrane lipid metabolic process, cellular homeostasis, and cell death, are highly expressed under combined treatments of ACh and ENK (Figure 3).

### Hemocyte Apoptosis After Neurotransmitter Treatment

**Figure 4:**
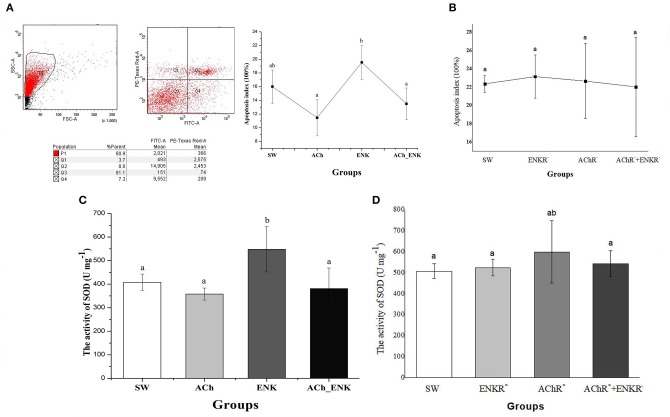
Determination of four key immune-related molecule expression. **(A)** Hemocyte apoptosis rate after LPS stimulation and treatments of ACh and ENK. **(B)** Hemocyte apoptosis rate after LPS stimulation and treatments of ACh and ENK when AChRs and ENKRs were blocked by antagonists. **(C)** The SOD activity after LPS stimulation and treatments of ACh and ENK. **(D)** The SOD activity after LPS stimulation and treatments of ACh and ENK when AChRs and ENKRs were blocked by antagonists.

To further validate the immunomodulation function of ACh and NE, the apoptosis index of oyster hemocytes after neurotransmitter stimulation was determined by flow cytometry (Figure 4). The apoptosis index of oyster hemocytes in ACh and ACh_ENK groups did change significantly after stimulation (*p* > 0.05), while it increased from 16.0 to 19.5% (Figure 4A, *p* < 0.05) in the ENK group compared with that in the SW group. However, when the ENKRs and AChRs were blocked by specific antagonists, no significant change of the apoptosis index was observed after ACh and ENK treatments (Figure 4B).

### The Effects of Neuroendocrine Regulation on SOD Activities

The activity of SOD was determined after LPS stimulation and neurotransmitter treatments (Figure 4). In the ENK group, SOD activity increased significantly from 408 to 548 U mg^−1^, compared with that in the SW group (Figure 4C, *p* < 0.05). No obvious change of SOD activities was observed in ACh and ACh_ENK groups (Figure 4C, *p* > 0.05). After the ENKRs and AChRs were blocked by specific antagonists, no significant change of SOD activity was observed after ACh and ENK treatments (Figure 4D).

### The Expression Changes of Several Important Immune-related Genes Under Neuroendocrine Regulation

**Figure 5:**
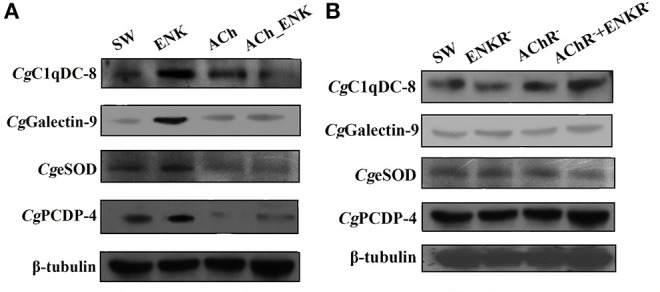
Determination of four key immune-related molecule expression. **(A)** The protein expression of *Cg*C1qDC-8, *Cg*Galectin-9, *Cg*eSOD, and *Cg*PCDP-4 after LPS stimulation and treatments of ACh and ENK. **(B)** The protein expression of *Cg*C1qDC-8, *Cg*Galectin-9, *Cg*eSOD, and *Cg*PCDP-4 after LPS stimulation and treatments of ACh and ENK when AChRs and ENKRs were blocked by antagonists.

The expression of four differentially co-expressed molecules in all three treatment groups were determined by Western blot assay. ENK treatment could induce the expression of *Cg*C1qDC-8, *Cg*Galectin-9, *Cg*eSOD, and *Cg*PCDP-4, while ACh treatment could significantly repress the expression of *Cg*eSOD and *Cg*PCDP-4. In addition, combined treatment of ACh and ENK could significantly inhibit the expression of *Cg*C1qDC-8, *Cg*eSOD, and *Cg*PCDP-4 upon LPS stimulation (Figure 5A). However, when the ENKRs and AChRs were blocked by antagonists, expression variation of these molecules could no longer be observed (Figure 5B, Table 1).

**Table 1:** Key differentially expressed proteins under ACh and ENK immunomodulation.

**Protein description**	**Protein accession number**	**Mean**		
		**ACh**	**ENK**	**ACh_ENK**
**IMMUNE RECOGNITION**				
Galectin-9	EKC40501.1	1.2655	1.3565	1.2755
Complement C1q tumor necrosis factor-related protein 3	EKC38694.1	1.6335	1.901	1.4295
**IMMUNE-RELATED ENZYME**				
Extracellular superoxide dismutase	EKC39002.1	1.763	1.7985	1.671
Peroxiredoxin-6	EKC23867.1	0.6645	0.6835	0.3355
**STRESS-RELATED PROTEIN**				
Universal stress protein A-like protein	EKC35480.1	0.7365	0.7875	0.5455
Neurogenic locus Notch protein	EKC40861.1	1.704	1.39	1.308
**APOPTOSIS**				
Programmed cell death protein 4	EKC31180.1	0.7855	0.83	0.552
**PHAGOCYTOSIS**				
Hemicentin-1	EKC40654.1	0.7015	0.705	2.36

## Discussion

The NEI regulatory network consists of nervous system, endocrine system, and immune system, which plays a reciprocal modulatory role in host homeostasis (10). Like vertebrates, molluscs also have evolved a less complex but complete NEI system, which uses neurotransmitters and hormones to regulate various immune processes (8). In our previous research, neurotransmitters ACh and ENK were found to play important roles in immune processes such as phagocytosis, apoptosis, and cytokine production in oyster *C. gigas* using transcriptomic methods (6). In the present study, iTRAQ and LC-ESI-MS/MS approaches were employed to further investigate the signaling pathways mediating the immunomodulation of ACh and ENK, as well as the combined modulatory effects of ACh and ENK.

Bivalves live on filter-feeding, and the biological environmental stressors such as microbial pathogens could be deadly threats (26). In order to survive from such challenging environment, bivalves have evolved a primitive but complete NEI system, which can be activated immediately to synthesize neurotransmitters upon stimulation and then conduct a series of immunomodulation to maintain homeostasis. In the present study, LPS, the major component of the gram-negative bacteria, was used to activate the neuroendocrine system (6). Meanwhile, ACh and ENK were injected respectively and in combination into the oysters, and hemocytes were collected for proteomic sequencing. The overall statistical analysis revealed the relative dispersion and parallelism between three repeats in each group, suggesting that the proteome comparison was of great statistical confidence. A total of 5,379 proteins were identified from 26,940 peptides, among which 25,865 were unique. These results showed overwhelming superiority of our workflow over the previous study using traditional 2D approach, which reported no more than 500 proteins or protein spots in this species (27, 28). The differentially expressed proteins in the neurotransmitter treatment groups (ACh, ENK and ACh_ENK) were further analyzed. A total of 2,590 proteins were uniquely differentially expressed in these three experimental groups, with 1,475 (57.0%) up-regulated and 1,115 (43.0%) down-regulated. The protein expression abundance analyses demonstrated that the proteome of oyster *C. gigas* hemocytes was remodeled under neurotransmitter regulation, indicating that the regulation of neurotransmitter was crucial for the immune response in oyster. Furthermore, there were 172 proteins differentially expressed in all three experimental groups compared with that in the SW group. The protein expression patterns in ACh and ENK groups were quite similar, while that in the ACh_ENK group showed an opposite style (Figure 1C). The results in Figure 2E also implied that the combined regulation of two neurotransmitters could revert the response pattern induced by a single kind of neurotransmitter. Previous transcriptomic studies reported that an injection of ACh after LPS stimulation could down-regulate the immune response level of oyster hemocytes, while ENK treatment induced opposite results (6). Besides, combined treatment of ACh and ENK could suppress the immune response level, which shared similar regulation modes of ACh (6, 19, 29). Thus, results in the present study suggested that the immunomodulation of both ACh and ENK could induce the differentially expression of many molecules just as indicated by the transcriptome data, and there were lots of common proteins shared by their regulation pathways. However, combined regulation of multiple neurotransmitters might result in totally different protein expression patterns and even revert the response pattern by single kind of neurotransmitter.

In order to explore the accurate immune regulation conducted by oyster hemocytes after LPS stimulation and neurotransmitter treatments, the enrichment of differentially expressed proteins in response to ACh, ENK, and ACh_ENK regulation was analyzed (Figure 2). In total, 529, 335, and 611 proteins were significantly up-regulated, while 370, 245, and 500 proteins were significantly down-regulated after ACh, ENK, and ACh_ENK treatments, respectively. There were 172 proteins differentially co-expressed in all three treatment groups, suggesting that they might be indispensable for the neuroendocrine modulation in oyster hemocytes. Among these proteins, a series of immune-related molecules were identified, including galectin-9 (EKC40501.1), complement C1q tumor necrosis factor-related protein 3 (EKC38694.1), extracellular SOD (EKC39002.1), peroxiredoxin-6 (EKC23867.1), universal stress protein A-like protein (EKC35480.1), neurogenic locus Notch protein (EKC40861.1), programmed cell death protein 4 (EKC31180.1), and hemicentin-1 (EKC40654.1). Further biological validation revealed that ENK treatment could induce the expression of *Cg*C1qDC-8 and *Cg*Galectin-9. When the ENKRs were blocked by specific antagonists, no significant changes of *Cg*C1qDC-8 and *Cg*Galectin-9 expression was observed after ENK treatments. Galectins and C1qs are critical molecules for immune recognition in innate immunity. Galectin is a large evolutionally conserved protein family, which is universally present in a wide variety of eukaryotic organisms ranging from fungi to mammals (30). Galectins in marine invertebrates have been proven to be involved in innate immune defense (31, 32). C1q domain-containing (C1qDC) proteins are another sort of important immune recognition molecules and act as PRRs in a molluscan immune system (33–36). A previous study demonstrated that the expression of PRRs as galectin might be modulated by TNF pathway. In mammals, Galectin-9 could inhibit TLR7-mediated autoimmunity in murine lupus models (37) and suppress the apoptosis in human rheumatoid arthritis synovial fibroblasts (38). Galectin-9 protein was overexpressed in astrocytes, and the apoptosis of encephalitogenic T-cell was promoted after TNF stimulation (39). It was reported that ENK could induce the expression of TNFs in oyster hemocytes (6). In the present study, ENK was found to induce the expression of *Cg*Galectin-9 and *Cg*C1qDC-8. It was hypothesized that ENK could prompt the immune regulation by enhancing the immune activities of oyster hemocyte against LPS stimulation through TNF pathway.

Apart from immune recognition, the redox equilibrium and apoptosis of oyster hemocyte were also found to be modulated by ACh and ENK upon LPS stimulation. Western blotting results showed that ENK treatment could induce the expression of *Cg*eSOD and *Cg*PCDP-4, while ACh treatment significantly repressed their expressions. When ENKRs and AChRs were blocked by their specific antagonists, the apoptosis index, SOD activity, and the expressions of *Cg*eSOD and *Cg*PCDP-4 did not change significantly after the treatments of ACh and ENK. SOD is one of the key enzymes in the cellular immunity of molluscs, and *Cg*eSOD is responsible for the redox equilibrium in oyster hemolymph (32, 40). The increased SOD activities demonstrated that the bivalves could produce more oxygen radicals to kill invasive pathogens (41, 42). Apoptosis is an important mechanism for the adequate clearance of infected, damaged, and exhausted cells, especially when the hosts suffer from infection or dissemination (43). Apoptosis was also reported to be one of the most efficient ways to programmably eliminate dead hemocytes in oyster, and *Cg*PCDP-4 was involved in hemocyte apoptosis. In our previous study, ENK was found to up-regulate the apoptosis index of oyster hemocyte, while ACh exerted opposite functions (6). Interestingly, combined treatment of ACh and ENK could significantly inhibit the expression of *Cg*C1qDC-8, *Cg*eSOD, and *Cg*PCDP-4 upon LPS stimulation, indicating that the immune regulatory functions of ENK might be overwhelmed by ACh, which was consistent with a previous study (6). The above results indicated that the immune system in oyster was activated after LPS stimulation, and ACh and ENK were possibly able to modulate the immune responses such as pathogen recognition, cell apoptosis, and redox equilibrium.

Furthermore, the network analysis of the differentially co-expressed proteins in response to neuroendocrine immunomodulation showed that several signaling pathways including ErbB signaling pathway, p53 signaling pathway, and FcγR pathway were highlighted within the combined immunomodulation of ACh and ENK. According to previous studies, the p53 signaling pathway could mediate cellular stress responses and initiate DNA repair, cell-cycle arrest, senescence, and, importantly, apoptosis (44–46). The EGF-R–ErbB signaling pathway is conserved in helminths and might be directly implicated in host–parasite molecular interplay (47–49). The antimicrobial responses of phagocytes are triggered by the cell surface receptors that recognize either conserved patterns on the surface of microorganisms or opsonins to coat the microorganisms (50), which requires the involvement of FcγR (51–53). These results suggested that ACh and ENK regulated the immune response in oyster by influencing host–pathogen interaction and hemocyte apoptosis, which was consistent with a previous report (6).

**Figure 6:**
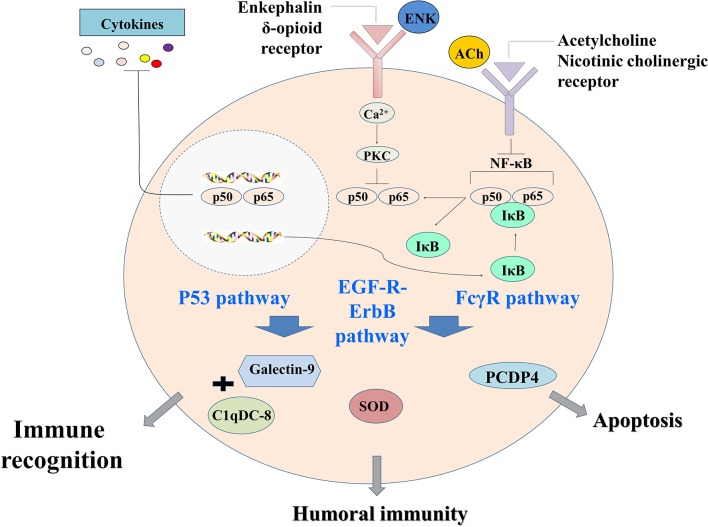
The neural immunomodulation patterns of oyster hemocytes at protein level after LPS stimulation.

In conclusion, a primitive but complete neuroendocrine immune regulatory network was revealed in oyster by proteomic analysis. It could be activated by LPS stimulation, and ACh and ENK were the two key neurotransmitters to regulate the immune response resulting from LPS stimulation. This immune regulatory network could possibly modulate immune recognition, redox equilibrium, and hemocyte apoptosis through p53 signaling pathway, EGF-R–ErbB signaling pathway, and FcγR signaling pathway (Figure 6). These results offered clues that different neurotransmitters might exert ordered immunomodulation through common signaling pathways in molluscs, and the NEI regulation was delicately controlled to maintain the homeostasis.

## Data Availability Statement

The datasets generated for this study can be found in the Dryad Data Repository, with doi 10.5061.

## Ethics Statement

All experiments involving animals reported in this study were approved by the Ethics Committee of the Institute of Oceanology, Chinese Academy of Sciences.

## Author Contributions

LS, LW, ZL, and ZZ conceived and designed the experiments. ZL and ZZ performed the experiments. ZL, ZZ, ML, and WW analyzed the data. LW and LS contributed reagents, materials, analysis tools. ZL, LW, ZZ, YZha, YZo, YZhe, and LS wrote the manuscript. All the authors read and approved the final manuscript.

## Conflict of Interest

The authors declare that the research was conducted in the absence of any commercial or financial relationships that could be construed as a potential conflict of interest.

## Supplementary Material

Figure S1 GO enrichment analysis of up-regulated expressed genes after LPS stimulation and ACh treatment. There were 529, 335, and 611 significantly up-regulated proteins, and 370, 245, and 500 significantly down-regulated proteins, identified in ACh, ENK and ACh_ENK groups, respectively.

Figure S2 GO enrichment analysis of up-regulated expressed genes after LPS stimulation and ENK treatment.

Figure S3 GO enrichment analysis of up-regulated expressed genes after LPS stimulation and combined treatment of ACh and ENK.

Figure S4 GO enrichment analysis of down-regulated expressed genes after LPS stimulation and ACh treatment.

Figure S5 GO enrichment analysis of down-regulated expressed genes after LPS stimulation and ENK treatment.

Figure S6 GO enrichment analysis of down-regulated expressed genes after LPS stimulation and combined treatment of ACh and ENK.

Table S1 Identification and relative abundance of haemocyte proteins in *Crassostrea gigas*.

Table S2 Description and relative expression levels of the 172 differentially co-expressed proteins after neurotransmitter treatments.

Table S3 Annotation of all the differentially expressed proteins after neurotransmitter treatments.

Table S4 GO terms for the *C. gigas* haemocyte proteome.
