# Supplementary figures and images for: A Signaling Pathway to Mediate the Combined Immunomodulation of Acetylcholine and Enkephalin in Oyster Crassostrea gigas
Source: Front Immunol. 2020 Apr 17;11:616. doi: 10.3389/fimmu.2020.00616 (PMC7180215; doi:10.3389/fimmu.2020.00616)

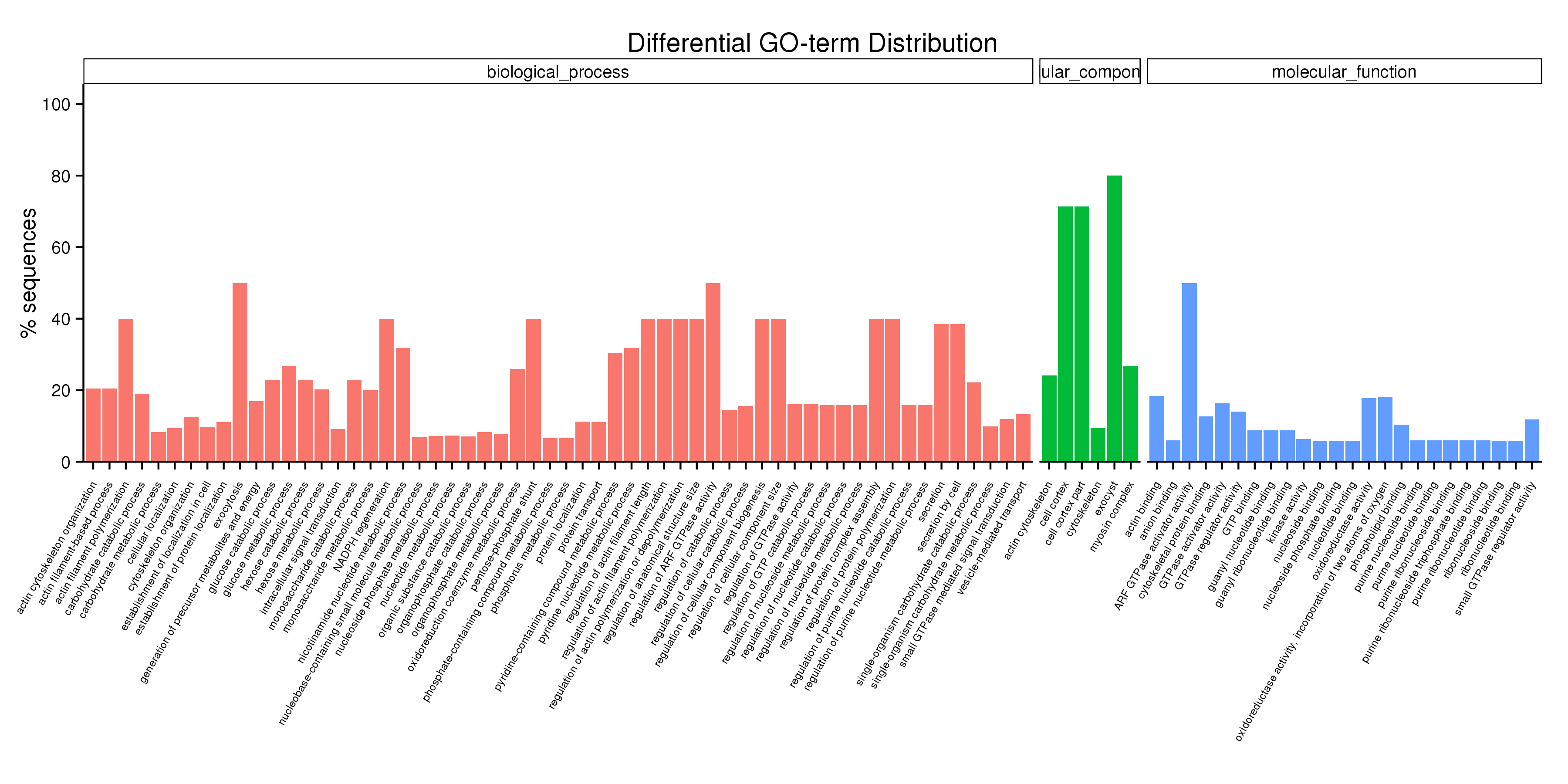

Supplement: Figure S1 — GO enrichment analysis of up-regulated expressed genes after LPS stimulation and ACh treatment. There were 529, 335, and 611 significantly up-regulated proteins, and 370, 245, and 500 significantly down-regulated proteins, identified in ACh, ENK and ACh_ENK groups, respectively. [file Image_1.JPEG]

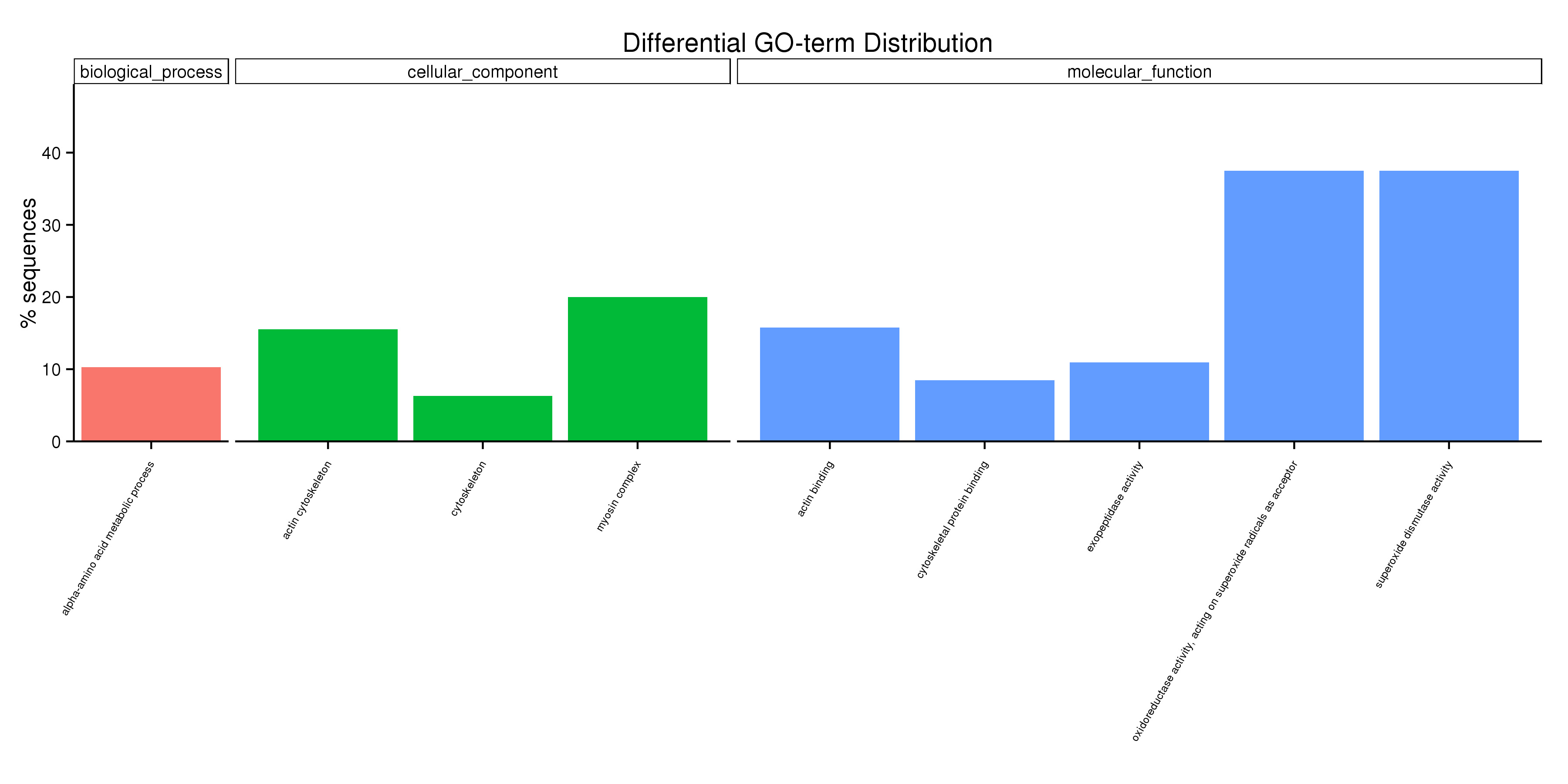

Supplement: Figure S2 — GO enrichment analysis of up-regulated expressed genes after LPS stimulation and ENK treatment. [file Image_2.JPEG]

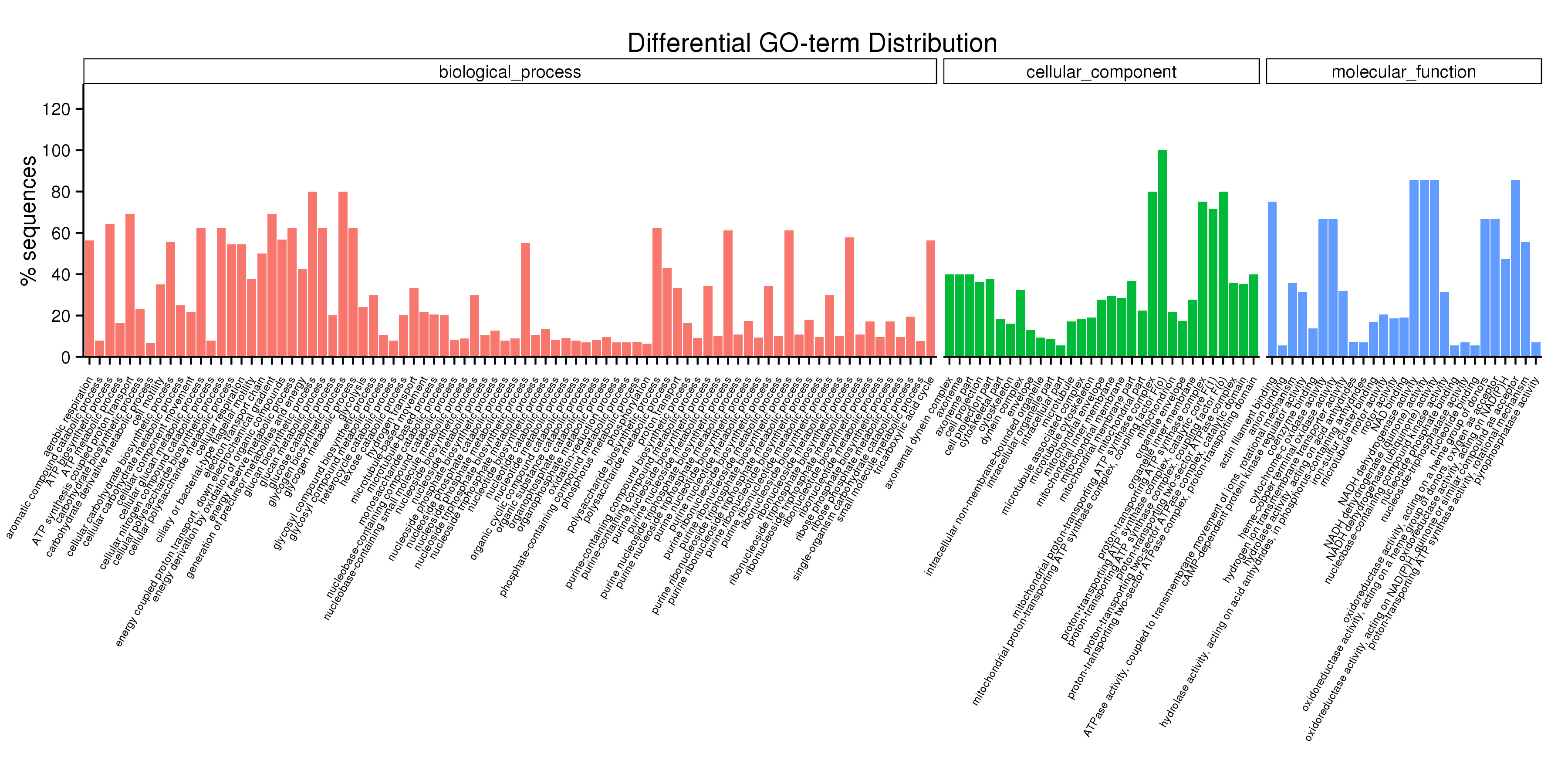

Supplement: Figure S3 — GO enrichment analysis of up-regulated expressed genes after LPS stimulation and combined treatment of ACh and ENK. [file Image_3.JPEG]

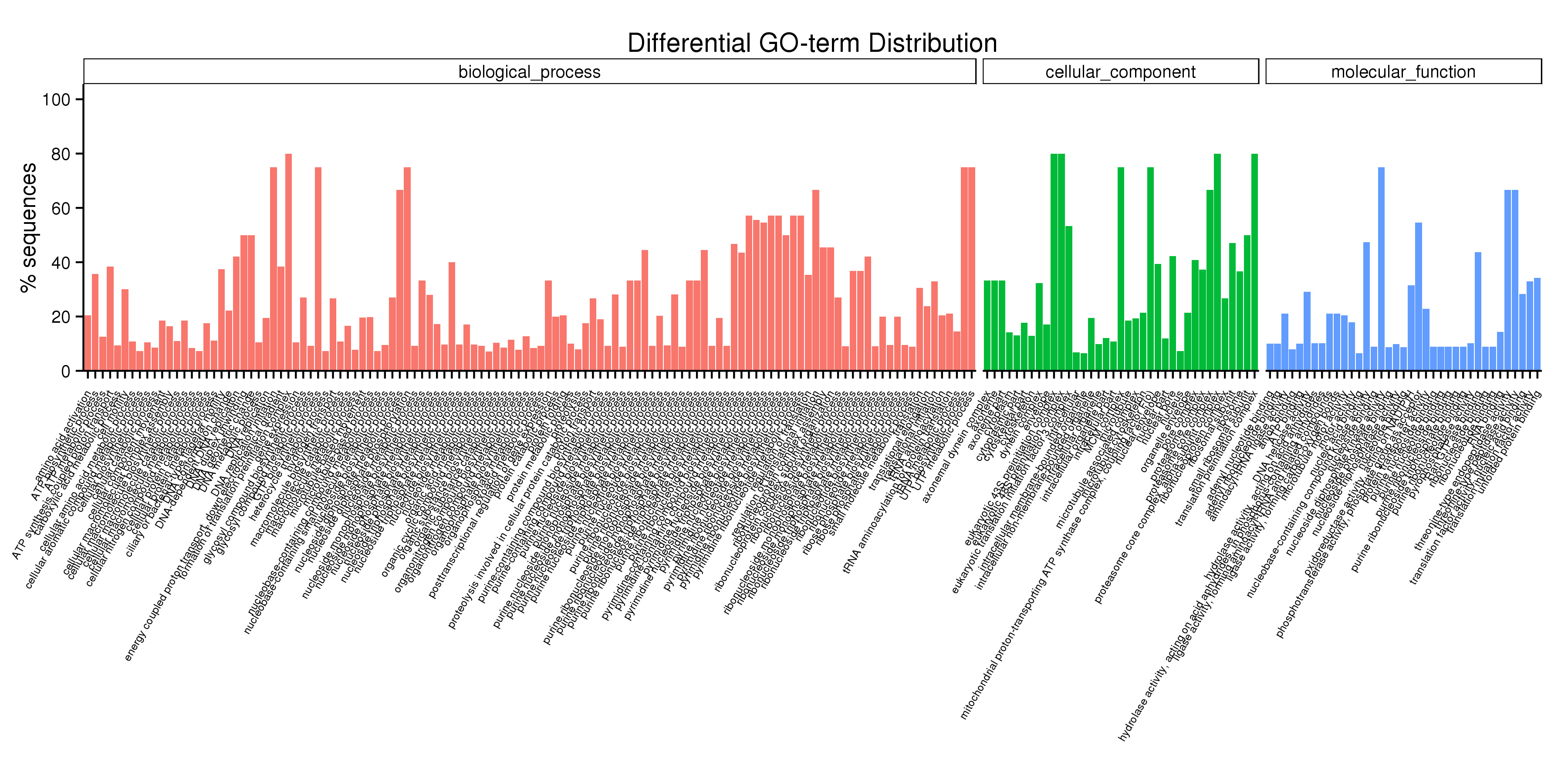

Supplement: Figure S4 — GO enrichment analysis of down-regulated expressed genes after LPS stimulation and ACh treatment. [file Image_4.JPEG]

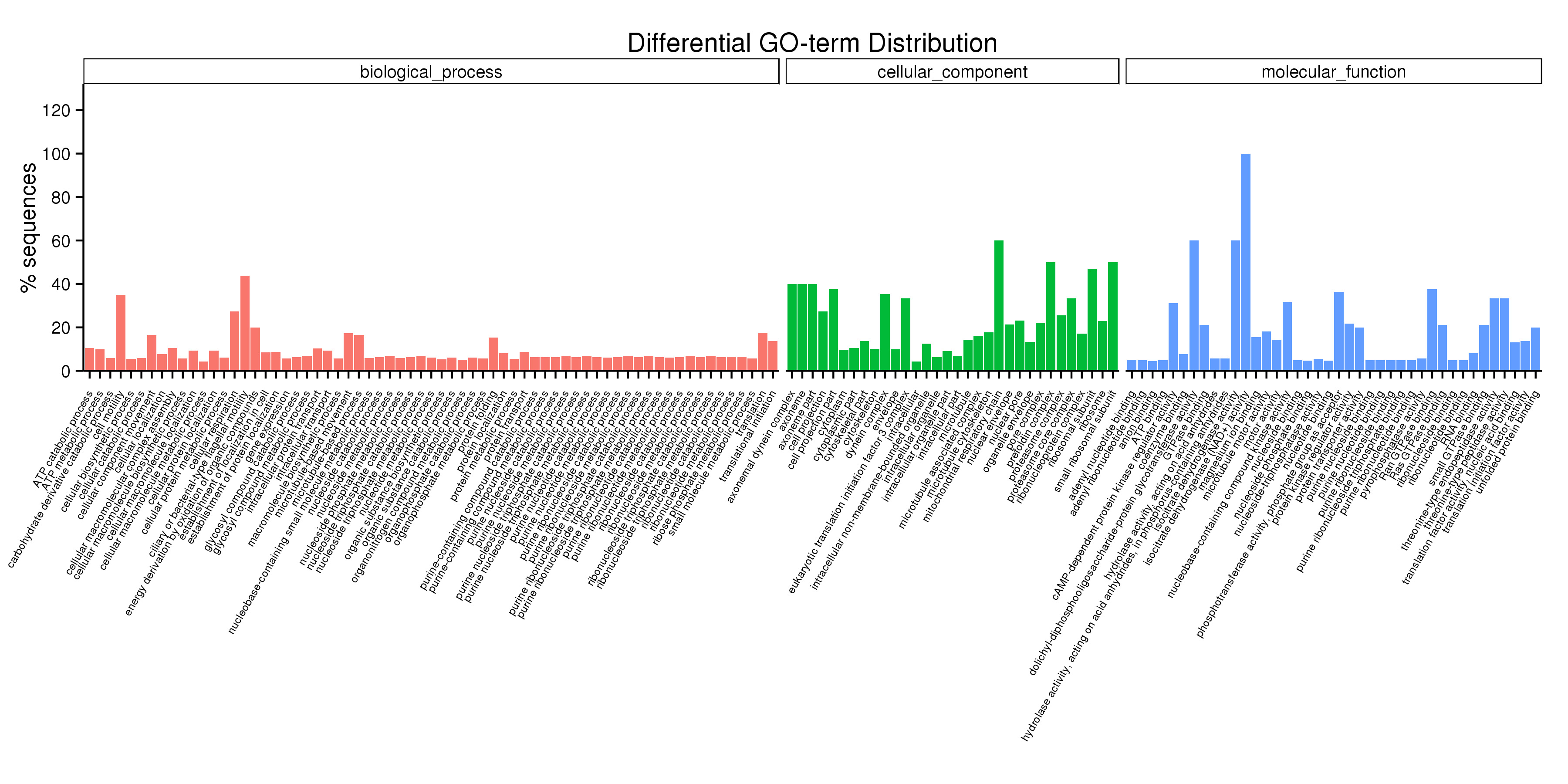

Supplement: Figure S5 — GO enrichment analysis of down-regulated expressed genes after LPS stimulation and ENK treatment. [file Image_5.JPEG]

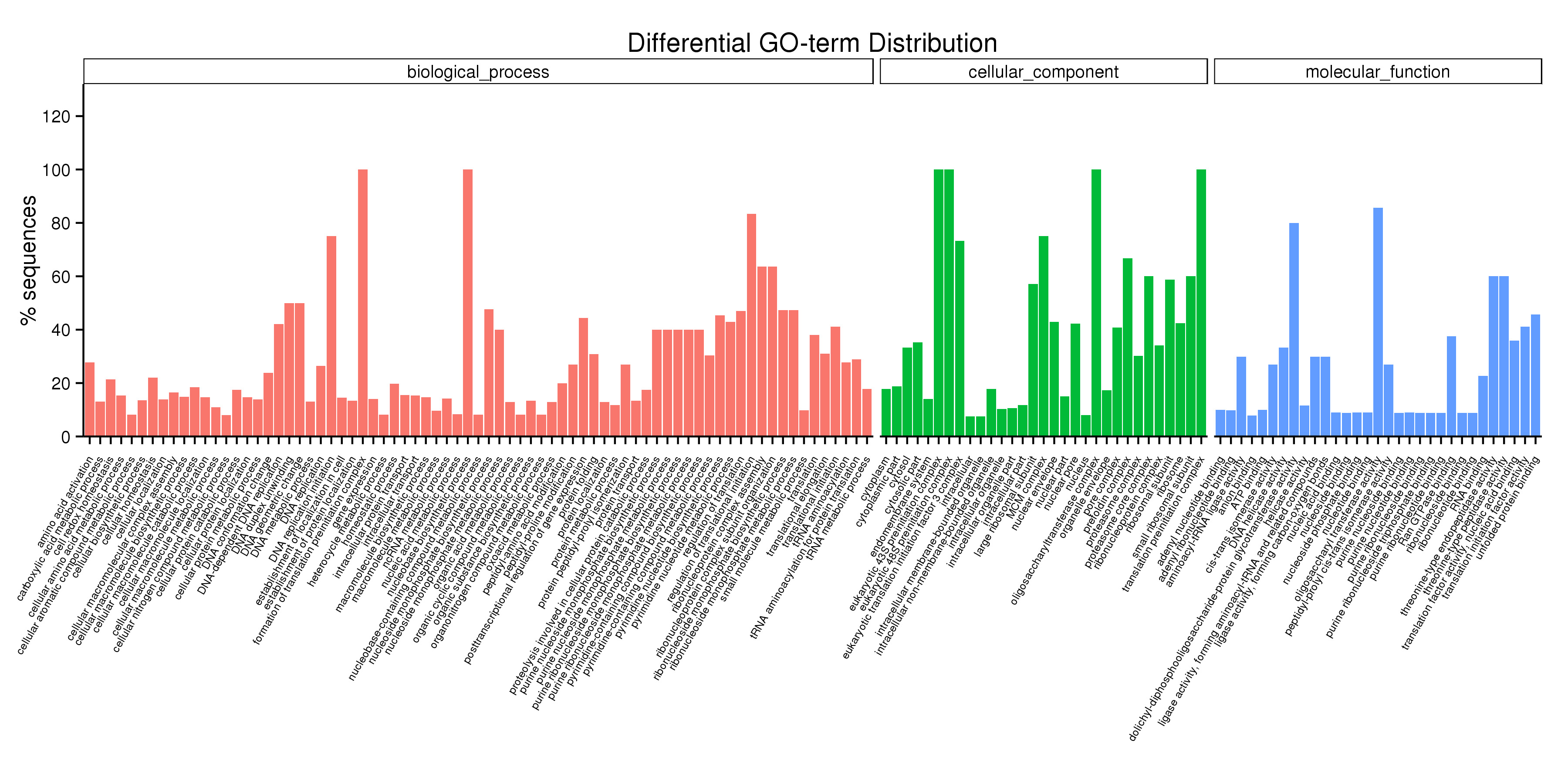

Supplement: Figure S6 — GO enrichment analysis of down-regulated expressed genes after LPS stimulation and combined treatment of ACh and ENK. [file Image_6.JPEG]
